# Portuguese Society of Ophthalmology and Portuguese Society of Human Genetics Joint Clinical Practice Guidelines for Genetic Testing in Inherited Retinal Dystrophies

**DOI:** 10.1111/cge.14691

**Published:** 2025-01-02

**Authors:** João Pedro Marques, Célia Azevedo Soares, Ana Luísa Carvalho, Sérgio Estrela‐Silva, Luísa Coutinho Santos, Lina Ramos, Eduardo Silva

**Affiliations:** ^1^ Departament of Ophthalmology Hospitais da Universidade de Coimbra (HUC), Unidade Local de Saúde (ULS) Coimbra, E.P.E. Coimbra Portugal; ^2^ Clinical and Academic Centre of Coimbra (CACC) Coimbra Portugal; ^3^ Clínica Universitária de Oftalmologia, Faculdade de Medicina Universidade de Coimbra (FMUC) Coimbra Portugal; ^4^ Medical Genetics Department Centro de Genética Médica Dr. Jacinto Magalhães, Unidade Local de Saúde (ULS) Sto António, E.P.E. Porto Portugal; ^5^ Unidade Multidisciplinar de Investigação Biomédica, Instituto de Ciências Biomédicas Abel Salazar (UMIB/ICBAS) and Laboratory for Integrative and Translational Research in Population Health (ITR) Universidade Do Porto Porto Portugal; ^6^ Departamento de Ciências Médicas Universidade de Aveiro Aveiro Portugal; ^7^ i3S – Instituto de Investigação e Inovação em Saúde Universidade do Porto Porto Portugal; ^8^ Department of Medical Genetics Hospital Pediátrico de Coimbra, Unidade Local de Saúde (ULS) Coimbra, E.P.E. Coimbra Portugal; ^9^ Clínica Universitária de Genética Médica Faculdade de Medicina, Universidade de Coimbra (FMUC) Coimbra Portugal; ^10^ Departament of Ophthalmology Unidade Local de Saúde (ULS) São João, E.P.E. Porto Portugal; ^11^ Instituto de Oftalmologia Dr. Gama Pinto Unidade Local de Saúde (ULS) São José, E.P.E. Lisboa Portugal; ^12^ Centro de Responsabilidade Integrado de Oftalmologia Pediátrica (CRI‐OftaPed), Hospital Dona Estefânia Unidade Saúde Local (ULS) São José, E.P.E. Lisboa Portugal

**Keywords:** genetic counselling, genetic testing, guidelines, inherited retinal dystrophies, ophthalmic genetics

## Abstract

The Portuguese Society of Ophthalmology and the Portuguese Society of Human Genetics developed clinical practice guidelines to streamline genetic testing for inherited retinal dystrophies (IRDs), underlining the critical role of molecular diagnosis in enhancing patient care. Genetic testing is pivotal in diagnosis, genetic counselling, prognosis and access to clinical trials, and new gene‐specific therapies. These guidelines recommend genetic testing in all IRD patients and provide a detailed assessment of available testing methods, ensuring that genetic counselling is integrated into ophthalmic care. Essential to this process is the inclusion of at least one genetic counselling session to effectively communicate and discuss implications of test results with patients and families/carers. Key recommendations include cascade testing to identify at‐risk family members and standardisation of variant classification according to international criteria to ensure consistency in diagnosis and care. Ophthalmological follow‐up is generally prescribed at intervals of 1–2 years for adults and 6 months for paediatric patients, to monitor disease progression and complications. Paediatric considerations are addressed, reflecting the complexities and ethical concerns associated with testing minors. These guidelines aim to elevate diagnostic accuracy, guide therapeutic decisions and ultimately improve patient outcomes, marking a significant advance in the genetic management of IRDs.

## Graphical abstract

This study provides clinical practice guidelines to streamline genetic testing for inherited retinal dystrophies (IRDs), underscoring the critical role of molecular diagnosis in enhancing patient care. These recommendations aim to elevate diagnostic accuracy, guide therapeutic decisions, and ultimately improve patient outcomes, marking a significant advance in the genetic management of IRDs.

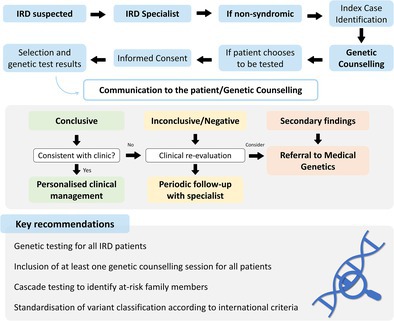

## 1 Introduction

Inherited retinal dystrophies (IRDs) are a group of genetic eye disorders marked by progressive retinal degeneration, potentially leading to irreversible vision loss [1, 2]. With a global prevalence of ~1 in 2000–3000 individuals, equating to more than 2 million people worldwide [3], IRDs collectively stand as one of the leading causes of blindness in the working‐age population. These conditions are genetically and phenotypically heterogeneous, with over 280 identified genes contributing to the vast spectrum of IRD presentations (Retinal Information Network [RetNet]) [4]. This diversity of IRD phenotypes poses a challenge for accurate clinical diagnosis. Disease onset can vary from birth to late adulthood [5], and whilst most IRDs are limited to the eye (non‐syndromic), up to 25% may present extraocular symptoms (syndromic) [6]. Clinical presentation varies greatly both within and between families, thus adding a further layer of complexity. Visual impairment can be very severe (e.g. legal blindness at birth in Leber Congenital Amaurosis) or almost unnoticeable (e.g. in cases of sector retinitis pigmentosa). Furthermore, IRDs can be associated with different inheritance patterns, including autosomal dominant, autosomal recessive, X‐linked, digenic or mitochondrial inheritance. Integrating genetic testing is crucial to enhance diagnostic precision and prognostic insight and offer information on inheritance risks and treatment prospects [5]. Despite advances in genetic technologies, the integration of these advances into the Portuguese healthcare system has proven uneven [7], a reality that significantly overlaps with other countries [8, 9]. Thus, a unified national approach to genetic testing for IRDs is currently an unmet medical need.

These clinical practice guidelines (CPGs) have been formulated to support healthcare professionals in the management of both adults and children with IRDs. The main goal is to establish a standardised approach to the optimal use of genetic testing for the diagnosis of IRDs. We aim to enhance diagnostic evaluations, improve patient access to genetic counselling, facilitate approved treatments, and promote participation in pivotal clinical trials. Although some considerations may be region‐specific, we believe this manuscript is of interest both to geneticists and ophthalmologists across the world, as it defines how to translate knowledge and technology into real‐world health gains through standardised practices adjusted to a limited resource scenario.

## 2 Methods

A steering committee of Portuguese geneticists and ophthalmologists selected for their extensive expertise in ophthalmic genetics defined the CPGs scope and formulated initial Patient/Population, Intervention, Comparison, and Outcomes (PICO) questions. After reaching consensus on these questions through a virtual meeting, an extensive literature review identified recommendation topics to address research gaps. Two Delphi rounds were then conducted to reach consensus on these topics. The first round involved an online questionnaire for panel members, followed by a second‐round meeting to resolve non‐consensus topics through discussion and feedback on statement clarity and overlap. Voting on recommendations was restricted to expert panel members. Consensus was defined as 100% agreement amongst all panel members. Recommendations were based on available evidence or expert experience (Table 1).

**TABLE 1:** Expert recommendations on the management and genetic testing of IRD patients.

	Minimum	Desirable	Optional
Population	Individuals with a clinical diagnosis of IRD Carrier screening in preparation for family planning	Individuals with a presumed/suspected IRD without an established clinical diagnosis Asymptomatic family members of individuals with diagnosed IRDs	
Diagnostic exams^a^	BCVA Visual field testing OCT Colour fundus photography Fundus autofluorescence	Electrodiagnosis Microperimetry Colour vision Ultra‐widefield colour fundus photography and fundus autofluorescence	Contrast sensitivity Low‐luminance BCVA OCTA
Informed consent	Testing timeline Long‐term implications Secondary findings (if applicable)	Education resources Research opportunities	
Genetic tests: index case	Virtual multigene panel sequencing (WES‐based)	WES or WGS	
Genetic tests: family assessment	Sanger sequencing (family variants) CNV analysis – specific variant screening		
Genetic counselling	The patient and/or guardian should have access to two genetic counselling appointments, before and after having the test results^c^	Integration of ophthalmological care Collaboration with patient advocacy groups	
Variant report	Implement a classification system based on established criteria (e.g. ACMG/AMP) for assessing the pathogenicity of genetic variants Adhere to guidelines by ESHG and ACMG/AMP for standardised reporting practices Laboratories with quality accreditation (e.g. CLIA)	Consider ongoing clinical trials of gene therapies for various IRDs, including retinitis pigmentosa, choroideremia, and X‐linked retinitis pigmentosa	Laboratories should actively search and report secondary findings from a minimum list of genes and categories recommended by ACMG^b^
Follow‐up	Regular ophthalmological follow‐up: 1–2 years for adults and 6 months for paediatric patients	For unsolved cases: Automatic reassessment of variants every year Assessment of variants on a biannual basis	

Abbreviations: ACMG: American College of Medical Genetics and Genomics; AMP: Association of Molecular Pathology; BCVA: best‐corrected visual acuity; CLIA: Clinical Laboratory Improvement Amendments; CNV: copy number variation; ESHG: European Society of Human Genetics; IRD: inherited retinal dystrophies; OCT: optical coherence tomography; OCTA: OCT angiography; WES: whole exome sequencing; WGS: whole genome sequencing.

^a^ The identification of bilateral and highly symmetrical retinal changes is in favour of an IRD and should prompt genetic testing.

^b^ Only applicable for WES or WGS and if requested upon authorisation outlined in the informed consent.

^c^ Genetic counselling can be provided by a properly trained ophthalmologist in the index case and in other affected family members. In healthy family members, it is recommended that counselling be carried out by a medical geneticist.

## 3 Clinical Assessment of Patients With IRDs

A comprehensive diagnosis of IRDs demands a thorough clinical history and examination, underpinning both the decision for genetic testing and its interpretation. To establish a clinical diagnosis, medical history (comorbidities and/or systemic disorders, current medication intake and history of retinotoxic medication), family history, pedigree, and dietary and lifestyle habits should be combined with deep phenotyping including functional evaluations and multimodal retinal imaging [1, 10]. Pedigree analysis should extend across at least three generations and include second‐degree relatives, even if unaffected, to unveil familial relationships, highlight inheritance patterns or elucidate events that could have masked disease presentation [1, 10, 11].

Visual function assessments are used to better understand IRDs, monitor disease progression and evaluate the impact of interventions. The most frequently evaluated functional parameters include best‐corrected visual acuity (BCVA), contrast sensitivity, colour vision, visual field testing and electrodiagnosis. The latter aids in distinguishing primary defects in rod or cone photoreceptor pathways and may unveil pathognomonic changes associated with a certain condition/gene [11, 12].

Retinal imaging plays a key role in assessing the retinal structure. Standard colour or wide‐field fundus photography may be performed at the initial visit to document fundus findings and gather data to monitor progression over time [1]. Spectral‐domain (SD) or swept‐source (SS) optical coherence tomography (OCT) and fundus autofluorescence (FAF) can precisely track disease progression, even when visual acuity does not change significantly [10]. FAF should be performed, as much as possible, with a short wavelength excitation light and reduced illumination, to observe the distribution of ocular fundus fluorophores. This examination can reveal valuable diagnostic information regarding the topographic distribution and extent of retinal disease and may often allow conclusions regarding retinal function. OCT generates cross‐sectional images of the retina, retinal pigment epithelium and choroid, enabling a thorough characterisation of disease processes [1]. Recent techniques such as OCT angiography (OCTA), adaptative optics, functional magnetic resonance imaging, laser speckle flowgraphy and retinal oximetry can further improve diagnostic precision [12].

In cases of suspected syndromic IRD, patients should also be referred to other specialists for follow‐up of extraocular disease.

### 3.1 Modified Testing Regimens for Paediatric Patients

Diagnosing IRDs in paediatric patients presents unique challenges [13]. Establishing accurate visual field measurements often requires repeated tests to ensure dependable outcomes. Similarly, examinations such as electroretinography (ERG) may necessitate sedation to improve accuracy. In cases of sedation, ERG should be performed immediately after anaesthesia induction, as it can impact ERG waveforms [14]. The decision for sedation should be carefully considered in light of the informational value the exam provides at a specific developmental stage [1]. A child‐friendly alternative is the use of hand‐held OCT by a trained specialist [15].

Detecting IRD symptoms in very young children can be challenging. However, it is critical for parents and paediatricians to detect symptoms as early as possible, fostering ophthalmological assessments, referrals for genetic testing and access to suitable therapies and treatments within the optimal developmental period. Nystagmus, central/peripheral vision loss, dyschromatopsia and photophobia are important signs/symptoms to keep in mind [13].

## 4 Ethical and Legal Considerations Before Genetic Testing

### 4.1 Consent

Genetic counselling and informed consent ensure that individuals or their legal representatives make well‐informed and voluntary decisions regarding genetic testing. Consent is required from adults or guardians of minors under 16, or adults unable to make informed decisions. Given the unique sensory challenges faced by IRD patients, autonomous and informed decision‐making requires special considerations. Alternative communication methods for obtaining informed consent may be employed, including: (1) Audio recordings of the informed consent; (2) Braille versions of consent forms; (3) Large‐print documents with high contrast for individuals with residual vision; (4) The presence of impartial third‐party witnesses to verify the consent process; or (5) Use of assistive technologies, such as screen readers or magnification software. These adaptations aim to empower IRD patients in making informed decisions about genetic testing whilst adhering to legal and ethical standards. Moreover, it is important to explain how incidental findings will be handled and to offer the option to opt‐out of receiving certain types of results. Consent should be provided after appropriate pre‐test genetic counselling, with laws supporting consent withdrawal at any stage without impact on patient care or rights [16, 17].

In cases involving minors, their views should be considered based on their age and maturity, and when appropriate, their consent should also be sought [18]. The process of consent should be informed, voluntary and comprehensible, emphasising the understanding of the test purpose, procedures, outcomes, and implications for patients and their families [19]. This involves providing a detailed explanation in clear and accessible language to ensure that the patient/guardian fully understands the benefits, risks and potential findings of the test.

### 4.2 Privacy and Confidentiality

Privacy and confidentiality are crucial in genetic testing due to concerns about the use, potential misuse and disclosure of genetic data. These concerns are well‐founded, given the potential impact on personal and professional relationships, as well as the risks of stigma and self‐esteem issues. To mitigate these, the health professional providing genetic counselling should have a comprehensive discussion with the patient, emphasising the strict legal framework in place to safeguard patient data and privacy [18].

In Portugal, health information, including genetic data, is considered confidential [16, 20]. Access to this data is strictly regulated, ensuring that genetic test results are shared only with the patient/legal guardian unless an explicit written authorisation has been given to share the information with another recipient [16]. In addition, genetic information from studies involving carriers and healthy individuals cannot be included in the electronic health records system [16].

### 4.3 Genetic Testing in Minors

In children with IRD, incorporating genetic testing early in the diagnostic process is highly beneficial for achieving enhanced diagnostic accuracy. Multigene panel sequencing (MPS) yields a diagnostic success rate of 84.7% in paediatric IRD cases, underlining the utility of genetic approaches in these circumstances [21].

Nevertheless, the American Academy of Paediatrics (AAP) and the American College of Medical Genetics and Genomics (ACMG) recommend a careful approach to genetic testing in minors, mirroring practices in other medical diagnostic evaluations [22]. This approach underscores the importance of informing parents/guardians about the risks and benefits associated with genetic testing and securing their consent. Echoing these recommendations, the European Society of Human Genetics (ESHG) endorses genetic testing in asymptomatic minors, particularly for conditions manifesting in childhood. However, they stress that the pros and cons of such testing need careful evaluation, especially when no effective treatment or prevention strategies are available. In these instances, genetic testing might still be pursued if it offers psychological or social benefits for the child and their family [23].

The Portuguese law aligns with these international guidelines, specifying that genetic testing in minors is permissible only when it serves the direct benefit of the child. This requires informed consent from parents/guardians and, ideally, from the minor themselves. Furthermore, Portuguese legislation restricts predictive testing for diseases that typically onset until early adulthood, unless there is tangible evidence that early detection can lead to effective prevention or treatment of the condition in question [16].

## 5 Genetic Testing

**FIGURE 1:**
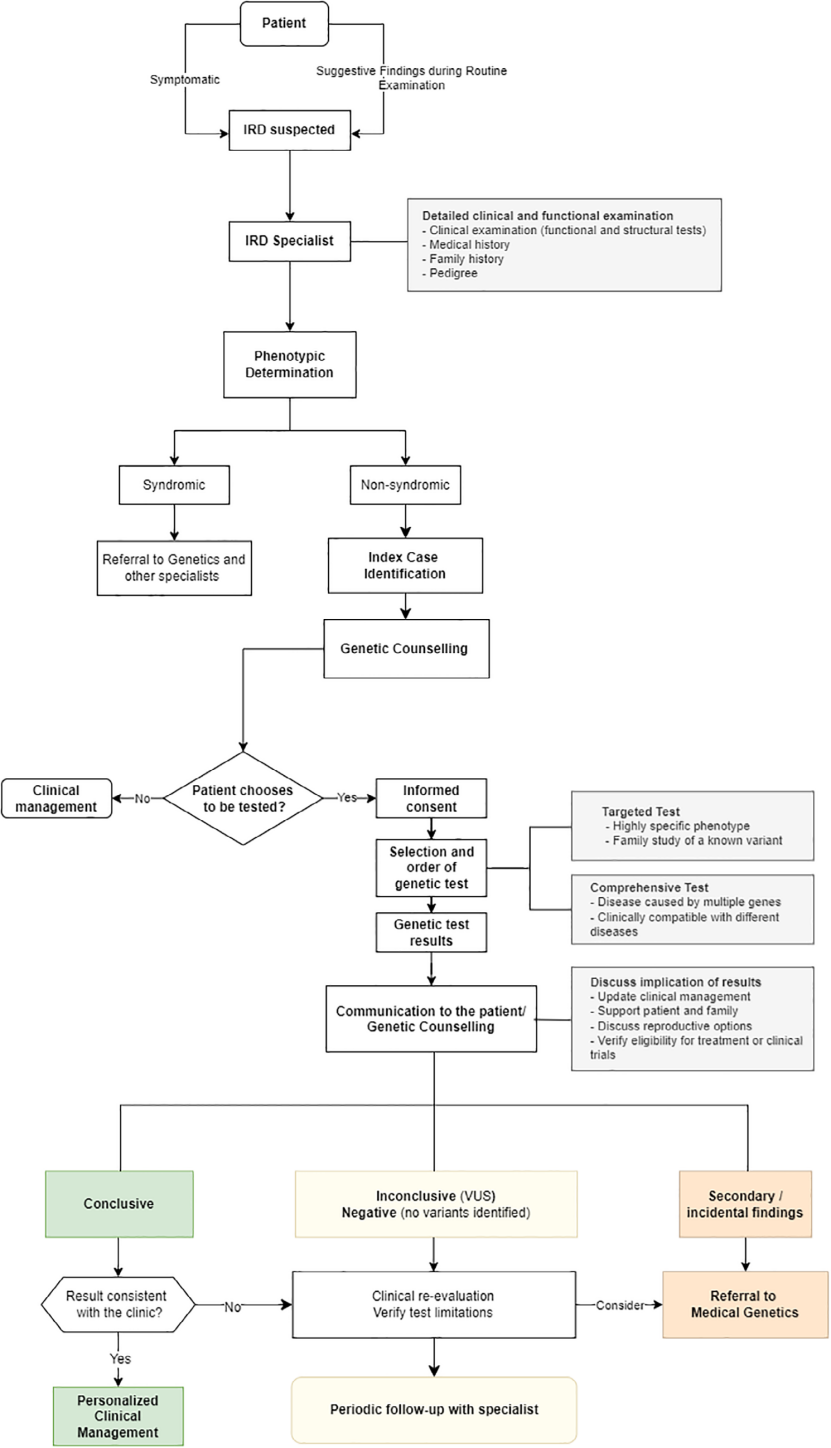
IRD management decision tree.

Following a detailed clinical assessment, it is essential to select the most appropriate genetic test to establish the diagnosis. Understanding the compatibility between genotype and phenotype is fundamental, as a single phenotype can result from mutations in different genes, and mutations in a single gene can lead to diverse clinical manifestations. The American Academy of Ophthalmology (AAO) highlights the importance of genetic testing for IRD, especially in cases indicative of Mendelian inheritance [24]. Genetic testing enables tailored therapeutic interventions thus advancing personalised medicine in ophthalmic care [25]. Moreover, the identification of disease‐causing gene variants allows a better understanding of the disorder and its inheritance patterns [18]. The decision tree for IRD management, as shown in Figure 1, provides a comprehensive guide for integrating clinical assessments and genetic testing protocols to optimise patient outcomes.

In accordance with Portuguese legislation [16, 17], genetic information can be obtained through a wide range of diagnostic methods, including molecular biology, cytogenetics, biochemistry, physiology, imaging tests or by collecting family information in the form of a family tree. This approach aligns with the AAO recommendations [24], which emphasise the importance of a comprehensive patient assessment for informed decision‐making and personalised care.

For ophthalmologists focusing on IRDs, familiarity with legal requirements is crucial for the ethical use of genetic testing. Online resources such as OMIM (Online Mendelian Inheritance in Man), RetNet and ClinVar offer valuable databases that help correlate specific genetic mutations with their clinical manifestations, guiding the choice of genetic testing and improving diagnostic accuracy.

### 5.1 Selection of Candidates for Genetic Testing

The process of selecting candidates for genetic testing in the context of IRDs requires a systematic approach that integrates clinical diagnosis and an assessment of familial genetic history. This strategy aligns with the principles of cascade testing [26], essential for early disease identification, and the provision of informed guidance for affected families.

#### 5.1.1 Clinical Diagnosis and Index Case Identification

The diagnostic process begins with the symptomatic family member, the index case. This individual serves as the starting point for genetic investigation and establishes a genetic basis for further familial investigation.

When requesting genetic testing for the index case, the referral to the laboratory should include comprehensive clinical and familial information to aid variant interpretation. This includes the patient's diagnosis details (including disease onset and progression), phenotype information from organs or systems that may be relevant to the diagnosis when a syndromic disease is suspected, identification of affected relatives, consanguinity disclosure, benefits of the genetic test and the patient's preference regarding being informed of potential secondary findings.

#### 5.1.2 Family Assessment and Cascade Testing

Following the diagnosis of the index case, it is crucial to conduct a thorough assessment of the familial genetic background. If a harmful genetic variant that correlates with the patient's symptoms is already known, it is invaluable to extend the exploration through cascade testing. This process involves screening family members at risk for specific mutations, thereby providing essential information about the presence and pattern of the disease within the family.

##### 5.1.2.1 Testing Sequence for Family Members

- *Immediate family cases*, particularly first‐degree relatives should be prioritised for genetic testing following mutation identification in the index case. This assesses their risk of developing the disease or transmitting the mutation. The decision of who to test next is informed by the mutation's inheritance pattern and its relevance to each family member's clinical scenario.
- *Healthy family members* at risk of being carriers of disease‐causing mutations may be recommended for genetic testing if there is evidence of analytical and clinical validity, and a clear clinical benefit. Personal autonomy, confidentiality and privacy of genetic information should be respected. The goal is to provide precise diagnoses, confirm inheritance patterns, aid in predicting visual function, guide family planning and facilitate early access to clinical trials. Of note, by the Portuguese law, testing in healthy individuals can only be performed by a clinical geneticist.
- Genetic testing in *minors* should be approached cautiously, focusing on conditions where early intervention could alter disease outcomes in direct benefit to the minor. Testing for asymptomatic adult‐onset conditions is usually postponed until legal adulthood unless immediate benefits are evident.
*Prenatal testing and pre‐implantation genetic testing (PGT)* are intended to detect early‐onset conditions or diseases that significantly affect health, focusing on disease prevention and addressing disabilities within legally defined timeframes. These must have a clear medical rationale and should not be used for the sole purpose of informing parents. Prenatal genetic testing for IRDs in Portugal is governed by Law 16/2007, which provides a legal framework for pregnancy termination under specific circumstances. According to this law, termination is permissible if there are reliable grounds to predict that the unborn child will suffer from an incurable, severe disease or congenital malformation. The procedure is performed within the first 24 weeks of pregnancy. The decision‐making process involves a local institutional technical committee that reviews each case and assesses the specific IRD's risk, severity and quality of life impact. PGT in Portugal is regulated under Law No. 32/2006, which provides the legal framework for medically assisted reproduction. The National Council for Medically Assisted Procreation (CNPMA) oversees PGT and periodically updates guidelines to ensure compliance with ethical and legal standards.

##### 5.1.2.2 Strategic Selection in the Absence of Prior Family Testing

In situations lacking prior family testing or if results are inconclusive, thoughtful consideration is required to determine which family member should undergo testing first. Priority should be given to available and clinically affected members. In suspected X‐linked recessive diseases, testing a male could yield clearer insights due to the simplicity of their X chromosome makeup.

### 5.2 Choosing the Appropriate Genetic Testing Method

Selecting the appropriate genetic testing method for IRD diagnosis is pivotal to make an accurate diagnosis, identify causative genes, and inform patient prognosis and management. This decision requires a detailed evaluation of testing modalities, considering their scope, potential for secondary findings, advantages, disadvantages and their suitability for specific clinical scenarios (Table 2).

**TABLE 2:** Overview of genetic testing methods for the molecular diagnosis of IRDs.

Testing method	Scope	Secondary findings	Advantages	Disadvantages	Suitable for
Sanger sequencing (SS)	Tailored to specific genes or regions	Possible if analysis designed to include them	Highly accurate for targeted regions; Customizable to specific research or clinical needs	Limited scope; higher cost per gene; not suited for large‐scale variant discovery	Confirming specific variants; investigating familial mutations
Virtual multigene panel sequencing (WES‐based)	Customizable sequencing based on WES data	Customizable depending on analysis scope	Allows re‐analysis and inclusion of genes initially missed; Cost‐effective for targeted analysis; Enables broader genetic variant discovery within the exome	Limited to exonic regions May miss intronic and structural variants Dependent on initial WES quality and depth	Targeted analysis when WES data available
Targeted multigene panel sequencing (MPS)	Specific gene panels	Intermediate probability	Customizable gene targeting; Diagnosis of heterogeneous or overlapping diseases; Possibility screening introns; Targeted regions sequenced with a greater read and depth	Limited by panel selection; May miss non‐panel variants	Diseases with known gene mutations
Whole exome sequencing (WES)	Protein‐coding regions	High probability	Identifies coding variants; Potential for new gene discoveries	Misses intronic/structural variants; Ethical concerns	Broad variant discovery when phenotype is unclear
Whole genome sequencing (WGS)	Entire genome	Highest probability	Comprehensive analysis; Intronic/exonic variant identification	Complex data analysis; Ethical concerns; Cost	Comprehensive diagnosis when other methods fail

Sanger sequencing (SS) offers unparalleled accuracy for targeted regions, making it ideal for disorders suspected to stem from a single gene mutation, such as X‐linked retinoschisis. It is also useful for confirming suspected variants, investigating familial mutations, or for genes not well covered by broader sequencing methods [27]. Next‐generation sequencing (NGS) techniques, including targeted MPS, whole exome sequencing (WES) and whole genome sequencing (WGS), allow for the simultaneous screening of multiple genes or the entire genome. MPS focuses on specific gene panels, providing a middle ground in the probability of discovering incidental findings. It is particularly useful for diseases with known gene mutations, offering a detailed analysis of targeted regions; however, it is limited by panel selection, potentially missing variants in genes not included in the panel. WES targets protein‐coding regions and bridges the gap between targeted panels and whole genome analysis, uncovering coding variants with potential for novel gene discoveries. WGS is the most comprehensive method as it covers the entire genome, including intronic, exonic and intergenic regions [28]. Phenotype‐driven virtual panels using WES data are also a relevant option and can significantly increase diagnostic rates, providing a customisable and cost‐effective approach [29].

The choice between these methods depends on several factors like clinical scenario, suspected genetic aetiology and the extent of analysis required. Whilst MPS offers a cost‐effective, focused approach for well‐characterised conditions, WGS provides the most extensive coverage at a higher cost and complexity. It is important to note that coverage values for WES are not universal and can vary significantly between different laboratories. Therefore, it is advisable to contact the specific laboratory to obtain detailed information about their analysis. The development of web‐based tools like WEScover, which aids in determining whether to use WES or MPS testing, could be instrumental for ensuring comprehensive coverage of genes relevant to eye diseases [30].

Despite their value in diagnosing IRDs with extensive genetic heterogeneity or syndromic conditions, NGS‐based methods raise concerns about data analysis reliability, identification of variants of uncertain significance (VUS) and the ethical implications of incidental and secondary findings, if applicable. The ESHG recommends an initial targeted sequencing approach to minimise unsolicited findings, suggesting that genome‐wide analyses should only be justified based on clinical necessity and patient benefit [31]. This approach is particularly relevant when diagnosing children with probable genetic disorders, where genome‐scale sequencing is advised only after targeted sequencing fails to identify a causative variant. Structural variants are a prevalent source of genetic variation that has been implicated in many genomic disorders, including IRDs [32]. Although the annotation of point mutations (SNVs) and small insertions or deletions (indels) from WES data is standard, the annotation of copy number variation (CNV) is not [33]. Given the high frequency of CNVs in IRD‐associated genes, CNV screening is extremely relevant.

With the rapid advancement of technology, emerging innovations are poised to significantly enhance the diagnostic yield for IRDs. Two notable examples of these cutting‐edge technologies are long‐read sequencing [34] and optical genome mapping [35], both showing great promise to revolutionise IRD diagnostics.

Long‐read sequencing can provide more comprehensive coverage of complex genomic regions, improve the detection of structural variants and enhance the resolution of repetitive sequences [34].

Optical genome mapping allows for high‐resolution visualisation of genomic structure. This method can detect large structural variations, including CNVs, inversions and translocations, which are often challenging to identify using conventional sequencing approaches [35]. Despite their immense potential, integration into clinical practice should follow a rigorous evaluation process. Only upon the successful demonstration of their effectiveness and clinical utility should these emerging technologies be considered for widespread adoption in IRD diagnostics.

## 6 Communication and Management of Genetic Test Results

### 6.1 Standards for Variant Interpretation

Genotype–phenotype correlations in IRDs allow for adequate genetic testing interpretation. Age of onset, type of symptoms and deep phenotyping are cornerstones in the process of variant selection in cases presenting interpretative challenges. Yet, with increased access to sequencing, milder presentations of several IRDs are now frequently uncovered, illustrating that most conditions belong to a phenotypic spectrum rather than one “classic” phenotype.

Variant classification is vital for an accurate genetic diagnosis as it influences management decisions, prognostication and family planning. The ACMG and the Association for Molecular Pathology (AMP) jointly devised a framework for variant classification [36], guiding assessment of variant pathogenicity or significance [37]. This system classifies variants into five categories: (i) benign (clear evidence that the variant is not associated with the disease); (ii) likely benign (the variant is unlikely to cause disease, 90% certainty); (iii) uncertain (VUS); (iv) likely pathogenic (the variant is likely to cause disease, 90% certainty); and (v) pathogenic (strong evidence that the variant is the cause of the disease) [37].

In Portugal, genetic test reports must adhere to these international standards. Multiple tools and databases are available to assist laboratories and clinicians in understanding the functional significance of genetic variants with respect to their potential effects on genes and diseases, such as the key reference OMIM database [38].

For VUS that align with the patient's phenotype, further genetic analysis and family studies are imperative for possible reclassification. Patients with VUS should be promptly referred to a medical geneticist for comprehensive revision, aiding in the reclassification process and enhancing patient management. Data interpretation is a continuously evolving discipline. Variants should not be discarded just because a single disease‐feature is absent or unusual features are present. Disease manifestations may emerge over time and/or may not be present when an individual is investigated at a single point in time. The term “reverse phenotyping” (re‐evaluating the clinical findings of the individual in light of a potential pathogenic genotype) illustrates the ongoing urgency of exchange between the diagnostic laboratory and the referring physician [33]. This is not infrequent in syndromic IRDs where extraocular manifestations may not be present or even overlooked by the ophthalmologist. As new phenotypical data become available or additional testing is conducted, inconclusive results may be revisited, ensuring patient care remains informed by the most current genetic insights.

### 6.2 Communication of Test Results

Communicating genetic test results is a sensitive and critical step in patient care. In Portugal, the communication process is guided by the principles set out in Law no. 12/2005, mandating that patients receive their genetic information directly from healthcare providers during a medical consultation [16]. This approach facilitates an immediate and clear discussion about the results, their implications and the next steps, whilst also allowing patients or their legal guardians to ask questions, ensuring a comprehensive understanding of the genetic findings.

The genetic test report must be clear, concise, accurate and fully interpretive, addressing the clinical question authoritatively [39]. Genetic reports must include detailed information about the genetic variant's classification and its potential clinical significance according to ACMG standards. Portuguese guidelines also require validation by a qualified professional and inclusion of patient and physician identification, sample details, relevant clinical and family information, methodologies used, results and their interpretation, recommendations and the report date, ensuring standardised genetic reporting [Portaria no. 91/2024/1, Article no. 5]. Healthcare providers must deliver and explain these reports thoroughly to patients, including incidental and/or secondary findings that may have been discovered, as agreed during consent. The language must be intelligible to a broad audience, including patients and healthcare professionals from different specialties. At all times, patient confidentiality must be safeguarded.

### 6.3 Management of Secondary and Incidental Findings

The management of secondary and incidental findings in genetic testing is a complex aspect of post‐test analysis, necessitating clear guidance and patient consent.

Secondary findings refer to pathogenic variants that, with the consent of the patient, are proactively searched for during data analysis, even though they do not pertain to the clinical question at hand. These findings often include variants in a set of genes known to be associated with highly penetrant genetic disorders where established interventions can prevent or significantly reduce morbidity and mortality [40]. This process is applicable to both WES and WGS, where broad genomic data is available for analysis.

On the other hand, incidental findings are those unexpected (likely) pathogenic variants that are discovered unintentionally. These variants are unrelated to the primary clinical query that initiated the test but may have implications for other medical conditions. The protocols for managing these findings apply not only to WES and WGS but also to MPS.

The management of both secondary and incidental findings requires a pre‐established plan that is communicated during pre‐test counselling, which must include information on the scope of the test and the type of results that may be expected [41]. Patients must give informed consent that specifically addresses their preferences regarding the receipt of secondary and incidental findings and should be provided with the option to opt‐out of receiving information about genetic conditions unrelated to their primary reason for testing. The significance of these findings and their potential implications should be thoroughly explained, ensuring patients have the necessary support to understand and act upon this information [16].

When handling incidental findings in minors, healthcare professionals face a myriad of ethical and legal challenges, with the child's welfare as the utmost priority. The focus is on how disclosing such information might impact the child's health, psychological well‐being and potential access to treatments or preventative interventions. It is essential to obtain informed consent from parents/legal guardians [16], and to actively involve the minor in the decision‐making process as much as possible. Minors and their guardians should be offered the choice to opt‐out of learning about incidental findings that do not immediately impact the child's health or that cannot be actioned upon until adulthood.

## 7 Genetic Counselling

Genetic counselling plays a pivotal role in guiding patients and their families through the complex process of genetic testing, offering crucial information and support to facilitate informed decision‐making regarding their healthcare and family planning [11]. In Portugal, the provision of genetic counselling is firmly established as a legal right and a crucial aspect of patient care [16, 17]. The national legal framework highlights the importance of informed consent, ensuring patients are thoroughly educated about the genetic testing process, including its purpose, benefits, risks and potential outcomes.

A critical juncture for genetic counselling occurs before any genetic testing is initiated, to ensure that the patient (or legal guardian) has all the information necessary to make an informed decision about testing.

Following the availability of genetic test results, it is recommended to conduct a second counselling session to discuss these findings with the patient. The healthcare provider should interpret the results in the context of the patient's and their family's medical history and guide on the subsequent steps, ensuring that information about prognosis, treatment options and further considerations is not only up‐to‐date but also tailored to the individual's condition. Properly trained ophthalmologists can provide this counselling, being key players in the continuous care of IRD patients. However, in cases of syndromic disease, it is advisable to refer to a specialised medical geneticist for thorough evaluation and counselling [16]. A detailed checklist of topics to be systematically addressed before and after genetic testing is outlined in Table 3.

**TABLE 3:** Genetic counselling checklist for before and after genetic testing.

Before genetic testing	After genetic testing
Evaluate patient and family medical history for risk factors and genetic predispositions. Explain the test's nature, benefits, limitations and procedures. Clarify testing types and what advantages and limitations they entail. Prepare patients for the potential discovery of VUS or secondary/incidental findings, explaining the implications and how such findings will be managed. Discuss ethical considerations and the pros and cons of testing minors. Explore broader implications of genetic testing, including privacy, insurance, and discrimination concerns related to genetic testing. Discuss the cost of testing and insurance coverage, addressing potential barriers to access and ensuring patients are aware of financial considerations. Signing informed consent form.	Clearly explain the results, covering conclusive, or inconclusive results and their clinical implications. Offer guidance on medical management, prevention, or treatment options. Discuss the availability and relevance of clinical trials for conditions without established treatments. Offer support to help process emotions and assist in decision‐making. If syndromic features are identified, facilitate referrals to appropriate specialists for further evaluation and care. Discuss how results affect family planning and implications for other family members. Assist in communicating the results and their implications to family members. Propose ongoing management and monitoring plans. Provide updates on new information affecting health management.

### 7.1 Implications for Family Planning

After receiving pathogenic genetic test results, patients should be guided about the procedures for family testing and specialised counselling on reproductive options, if interested. These options are highly personalised, considering the specific genetic diagnosis, inheritance pattern, family's religious and cultural beliefs, and personal values [18].

For minors undergoing genetic testing, a specialised counselling session is advisable as they approach legal adulthood, typically between the ages of 16 and 18. This guidance is tailored to their level of understanding, ensuring that they are fully informed about their reproductive choices and the genetic implications these choices may have on future generations.

### 7.2 Surveillance and Monitoring Recommendations

Clinical follow‐up visits are recommended for IRD patients every 1–2 years [24] to align with ongoing identification of new pathogenic genetic variants and the evolving nature of IRD conditions. Additionally, genetic re‐evaluation is advised every 2–3 years or based on scientific advancements or changes in the family's medical history, to refine the patient's diagnosis and management plan.

For paediatric patients with IRDs, a more frequent review schedule of every 6 months is advocated to closely monitor disease progression and address emerging needs. Such visits are essential for adjusting refraction, updating prescription glasses, incorporating low vision aids, treating any visually significant complications and ensuring support at school for their educational and social development.

### 7.3 Access to Emerging Gene Therapy‐Based Treatments and Ongoing Clinical Studies

The therapeutic landscape for IRDs is rapidly evolving, with cutting‐edge approaches like gene therapy, RNA‐based interventions, stem cell therapy, retinal prosthetics, pharmacological treatments, optogenetics and integrative combination therapies emerging as potential treatments. Due to the immune‐privileged nature of the eye and the continuing identification of causative genes, gene therapy is a particularly promising avenue for the treatment of IRDs [42].

A proof to this promise is the development and regulatory approval of voretigene neparvovec‐rzyl, a pioneering gene replacement therapy designed for patients with biallelic *RPE65* variant‐associated retinal degeneration. This treatment marks a significant milestone as the first therapy capable of decelerating or halting vision loss progression in this patient population [43]. It is currently being administered in several countries worldwide, including Portugal. A significant number of other gene therapies are being developed and tested for different types of IRD.

## Author Contributions

All authors developed the concept and study design of the consensus manuscript, participated in the consensus discussions, reviewed and approved the final manuscript.

## Conflicts of Interest

The authors declare no conflicts of interest.

## Data Availability

Data sharing not applicable to this article as no datasets were generated or analysed during the current study.
